# Supplementary material for: The Effects of Dietary Silybin Supplementation on the Growth Performance and Regulation of Intestinal Oxidative Injury and Microflora Dysbiosis in Weaned Piglets
Source: Antioxidants (Basel). 2023 Nov 7;12(11):1975. doi: 10.3390/antiox12111975 (PMC10669228; doi:10.3390/antiox12111975)
Supplement: Supplementary file 1 [file antioxidants-12-01975-s001.zip › Supplementary data.pdf]

Supporting information

Table S1. Ingredient and nutrient composition of the basal diet for trial 1 (as-fed basis)

	Prestarter	Starter
Ingredients, %		
Corn	16.44	21.81
Extruded corn	32.00	40.00
Soybean meal	14.00	17.50
Extruded soybean	11.50	6.00
Fish meal	5.60	3.00
Whey	15.00	5.00
Soybean oil	1.00	1.20
Bran	-	1.50
Dicalcium phosphate	0.40	0.60
Limestone ($CaCO_3$)	0.75	0.94
L-Lysine HCl	1.20	1.05
DL-Methionine	0.09	0.07
Threonine	0.27	0.23
Tryptophan	0.02	0.01
Salt	0.40	0.40
Choline chloride (60%)	0.05	0.05
Premix[1]	1.08	0.64
Zinc oxide	0.20	-
Analyzed nutrient content		
Crude protein, %	19.02	17.55
Dry matter, %	91.67	91.12
Calcium, %	0.94	0.91
Phosphorus, %	0.61	0.57
Calculated nutrient content		
ME[2], Kcal/kg	3400	3350
Crude protein, %	19.50	18.00
Calcium, %	0.80	0.70
Phosphorus, %	0.65	0.60
Lysine, %	1.30	1.15
Methionine, %	0.38	0.34
Threonine, %	0.76	0.68
Tryptophan, %	0.21	0.19

[1] Premix supplied per kilogram of diet: vitamin A, 35.2 mg; vitamin D_3, 7.68 mg; vitamin E, 128 mg; vitamin K_3, 8.16 mg; vitamin B_1, 4 mg; vitamin B_2, 12 mg; vitamin B_6, 8.32 mg; vitamin B_{12}, 4.8 mg; niacin, 38.4 mg; calcium pantothenate, 25 mg; folic acid, 1.68 mg; biotin, 0.16 mg; iron ($FeSO_4 \cdot H_2O$), 171 mg; manganese ($MnSO_4 \cdot H_2O$), 42.31 mg; copper ($CuSO_4 \cdot 5H_2O$), 125 mg; selenium (Na_2SeO_3), 0.19 mg; cobalt ($CoCl_2$), 0.19 mg; iodine ($Ca(IO_3)_2$), 0.54 mg.

[2] ME = metabolizable energy.

Table S2. Ingredient and nutrient composition of the basal diet for trial 2 (as-fed basis)

Ingredient	Content (%)	Nutrient levels	Content
Corn	19.04	Analyzed nutrient level	
Extruded corn	32.00	Crude protein, %	19.00
Soybean meal	22.50	Dry matter, %	91.70
Fish meal	5.60	Calcium, %	0.94
Whey	15.00	Phosphorus, %	0.61
Soybean oil	1.40	Calculated nutrient level	
Dicalcium phosphate	0.40	ME[2], Kcal/kg	3400
Limestone ($CaCO_3$)	0.75	Crude protein, %	19.50
L-Lysine HCl	1.20	Calcium, %	0.8
DL-Methionine	0.09	Phosphorus, %	0.65
Threonine	0.27	Lysine, %	1.30
Tryptophan	0.02	Methionine, %	0.38
NaCl	0.40	Threonine, %	0.76
Choline chloride (60%)	0.05	Tryptophan, %	0.21
Premix[1]	1.08		
Zinc oxide	0.20		
Total	100		

[1] Premix supplied per kilogram of diet: vitamin A, 35.2 mg; vitamin D_3, 7.68 mg; vitamin E, 128 mg; vitamin K_3, 8.16 mg; vitamin B_1, 4 mg; vitamin B_2, 12 mg; vitamin B_6, 8.32 mg; vitamin B_{12}, 4.8 mg; niacin, 38.4 mg; calcium pantothenate, 25 mg; folic acid, 1.68 mg; biotin, 0.16 mg; iron ($FeSO_4 \cdot H_2O$), 171 mg; manganese ($MnSO_4 \cdot H_2O$), 42.31 mg; copper ($CuSO_4 \cdot 5H_2O$), 125 mg; selenium (Na_2SeO_3), 0.19 mg; cobalt ($CoCl_2$), 0.19 mg; iodine ($Ca(IO_3)_2$), 0.54 mg.

[2] ME = metabolizable energy.

Table S3. The primers for quantitative real-time PCR

Gene[1]	Accession number	Primer sequence (5'-3')	Size (bp)
GAPDH	NM_001206359.1	F: GCTTGTCATCAATGGAAAGG<br>R: CATACGTAGCACCAGCATCA	86
CAT	NM_214301.2	F: CCTGCAACGTTCTGTAAGGC<br>R: GCTTCATCTGGTCACTGGCT	72
SOD1	NM_001190422.1	F: GAAGACAGTGTTAGTAACGG<br>R: CAGCCTTGTGTATTATCTCC	93
SOD2	NM_214127.2	F: GCTGAAAAAGGGTGATGTTA<br>R: CTATGATTGATGTGGCCTCC	81
GPX1	NM_214201.1	F: TCTCCAGTGTGTCGCAATGA<br>R: TCGATGGTCAGAAAGCGACG	104
GPX2	NM_001115136.1	F: AGCCCCACTGTGAAATTCTT<br>R: CGTAGAAGGACTTGGCAATG	131
GPX4	NM_214407.1	F: GATTCTGGCCTTCCCTTGC<br>R: TCCCCTTGGGCTGGACTTT	173
Nrf2	XM_005671981.3	F: GACCTTGGAGTAAGTCGAGA<br>R: GGAGTTGTTCTTGTCTTTCC	103
Keap1	NM_001114671.1	F: AGCTGGGATGCCTCAGTGTT<br>R: AGGCAAGTTCTCCCAGACATTC	100
HO-1	NM_001004027.1	F: GAGAAGGCTTTAAGCTGGTG<br>R: GTTGTGCTCAATCTCCTCCT	74
NQO1	NM_001159613.1	F: GGACATCACAGGTAAACTGA<br>R: TATAAGCCAGAGCAGTCTCG	68
FIS1	XM_021086263.1	F: CCAAAGGGAGCAAAGAGGAGCA<br>R: CCTGGTTGTTCTGTGGCTCTGT	132
DNM1	XM_021069575.1	F: GTAAACCGAAGCCAGAAGGACA<br>R: CAAGTGGCGATAGGAAGGGTGG	102
MFN1	NM_001315732.1	F: CAATAGAAGAGAGGGAAGACC<br>R: TATTTGCCACCTCCTCTGTAA	117
MFN2	XM_021095370.1	F: AGAGGAGAAGAGGAGCGTCAAGA<br>R: ACATCACACTCACCAGGCTGC	95
OPA1	XM_021070065.1	F: CAGAGGATGGTGCTTGTTGAC<br>R: AGTATGATGGCGTTGGGATTC	128
NDUFS2	XM_005663166.3	F: CTAAACGCGCAGAGATGAAGA<br>R: CCTCAATGGCAGTGTATGTGG	108
NDUFV2	NM_001097475.2	F: CCCAGATACTCCATTTGATTTCA<br>R: AATTTCTGCCACCTTGTTCATG	169
SDHA	XM_021076930.1	F: TCTCTGAGGCCGGGTTTAACACA<br>R: CACCTCCAGTTGTCCTCCTCCAT	124
UQCRB	NM_001185172.1	F: GGATGACGATGTAAAAGAAGCCA<br>R: TCCTCCTCATATTTTGTCCACTG	141
ATP5H	XM_021066093.1	F: CATTGACTGGGTAGCCTTTG<br>R: CTTCTCAGGTAGAGCAGCCA	115

MUC2	XM_021082584.1	F: CCGCATGGATGGCTGTTTCT<br>R: CATTGCTCGCAGTTGTTGGT	147

[1] GAPDH = glyceraldehyde-3-phosphate dehydrogenase; CAT = catalase; SOD = superoxide dismutase; GPX = glutathione peroxidase; Nrf2 = nuclear factor-erythroid2-related factor 2; Keap1 = kelch-like ECH-associated protein l; HO-1 = heme oxygenase-1; NQO1 = NAD(P)H: quinone oxidoreductase 1; FIS1 = fission; DNM1 = dynamin 1; MFN = mitofusin; OPA1 = mitochondrial dynamin-like GTPase; NDUFS2 = NADH ubiquinone oxidoreductase core subunit S2; NDUFV2 = NADH ubiquinone oxidoreductase core subunit V2; SDHA = succinate dehydrogenase complex flavoprotein subunit A; UQCRB = ubiquinol-cytochrome c reductase binding protein; ATP5H = ATP synthase subunit d; MUC2 = mucin 2.

Table S4. The list of antibodies used in western blotting.

Antibody[1]	Dilution	Company	Cat#
Rabbit polyclonal anti-cleaved-caspase3	1:1000	Affinity Biosciences	AF7022
Rabbit polyclonal anti-Bax	1:1000	Affinity Biosciences	AF0120
Mouse monoclonal anti-Bcl-2	1:1000	Abcam	ab117115
Rabbit polyclonal anti-ZO-1	1:1000	Invitrogen	61-7300
Rabbit polyclonal anti-Occludin	1:1000	Abcam	ab31721
Rabbit polyclonal anti-Claudin1	1:1000	Abcam	ab129119
Rabbit monoclonal anti-GAPDH	1:1000	CST[2]	2118
HRP-linked anti-mouse IgG	1:2000	CST	7076
HRP-linked anti-rabbit IgG	1:2000	CST	7074

[1] ZO-1 = zonula occludens-1; GAPDH = glyceraldehyde-3-phosphate dehydrogenase.
[2] CST = Cell Signaling Technology.

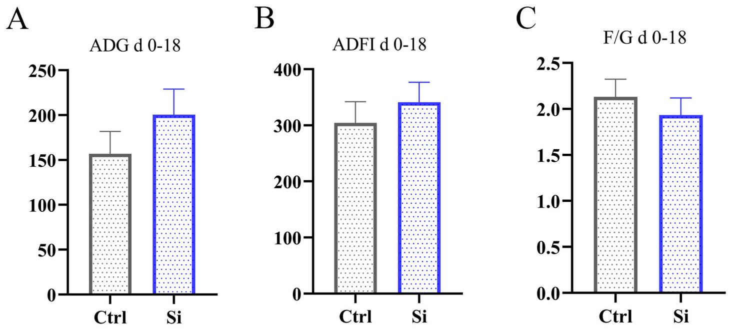

Figure S1. Effect of silybin on growth performance of piglets before the paraquat challenge. (A) ADG on days 0-18. (B) ADFI on days 0-18. (C) FCR on days 0-18. Data are expressed as mean ± SE. $^{*}p < 0.05$, $^{**}p < 0.01$. Ctrl, basal diet group treated with saline; Si, silybin diet group treated with saline; ADG = average daily gain; ADFI = average daily feed intake; F/G = ADFI/ADG.

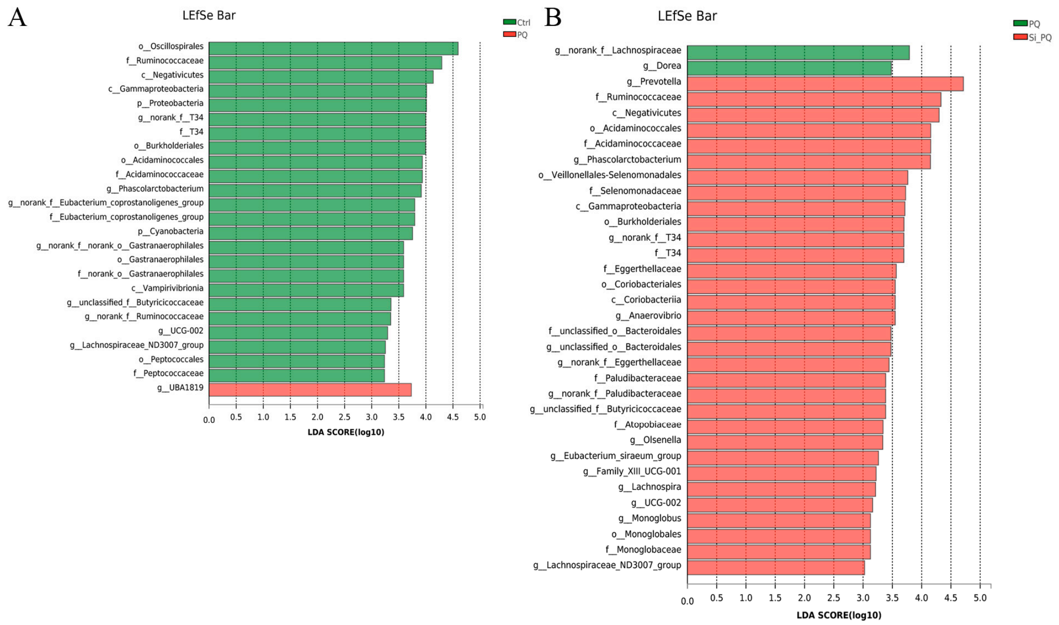

Figure S2. Histogram of linear discriminant analysis from genus to species level. (A) Ctrl vs. PQ. (B) PQ vs. Si + PQ. Ctrl, basal diet group treated with saline; Si, silybin diet group treated with saline; PQ, basal diet group treated with paraquat; Si + PQ, silybin diet group treated with paraquat; LEfSe = Linear discriminant analysis effect size.
