# Supplementary material for: The Effects of Dietary Silybin Supplementation on the Growth Performance and Regulation of Intestinal Oxidative Injury and Microflora Dysbiosis in Weaned Piglets
Source: Antioxidants (Basel). 2023 Nov 7;12(11):1975. doi: 10.3390/antiox12111975 (PMC10669228; doi:10.3390/antiox12111975)
Supplement: Supplementary file 1 [file antioxidants-12-01975-s001.zip › Supplementary File S1-The underlying raw images of Western blot/The underlying raw images of Western blot.pdf]

Supporting information

The underlying raw images of Western blot

For ZO-1:

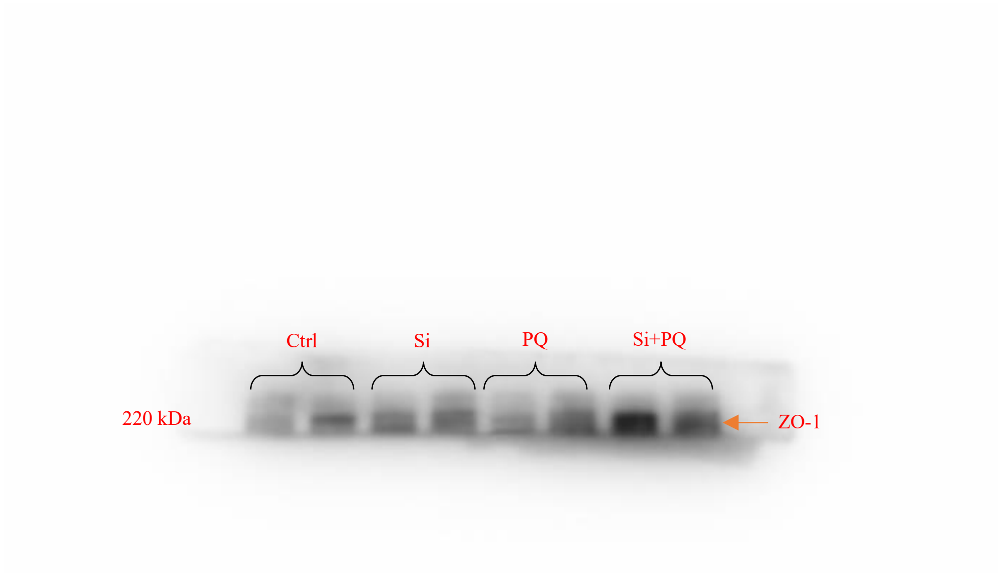

Fig. 1 Raw image of ZO-1 expression determined by Western blot (Repeat 1 and Repeat 2). Ctrl, basal diet group treated with saline; Si, silybin diet group treated with saline; PQ, basal diet group treated with paraquat; Si + PQ, silybin diet group treated with paraquat.

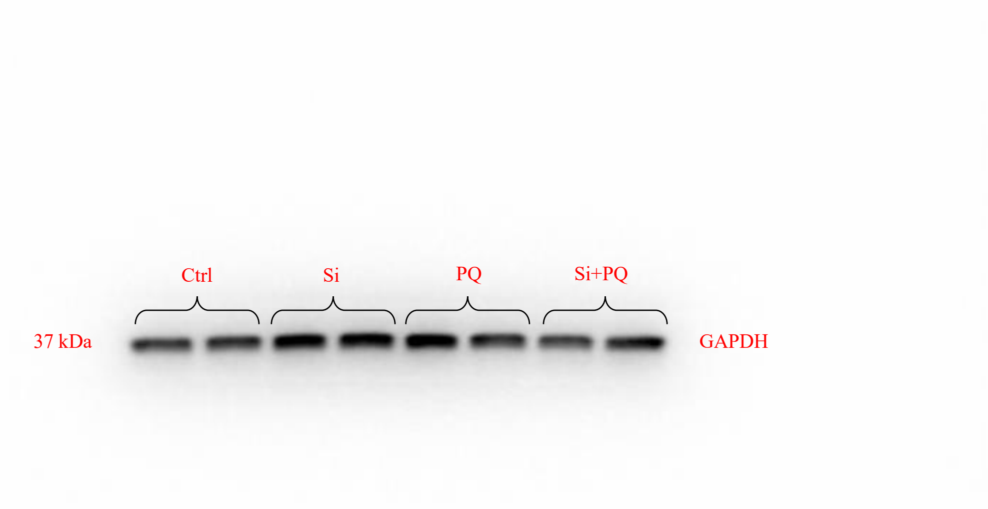

Fig. 2 Raw image of GAPDH expression determined by Western blot (Repeat 1 and Repeat 2). Ctrl, basal diet group treated with saline; Si, silybin diet group treated with

saline; PQ, basal diet group treated with paraquat; Si + PQ, silybin diet group treated with paraquat.

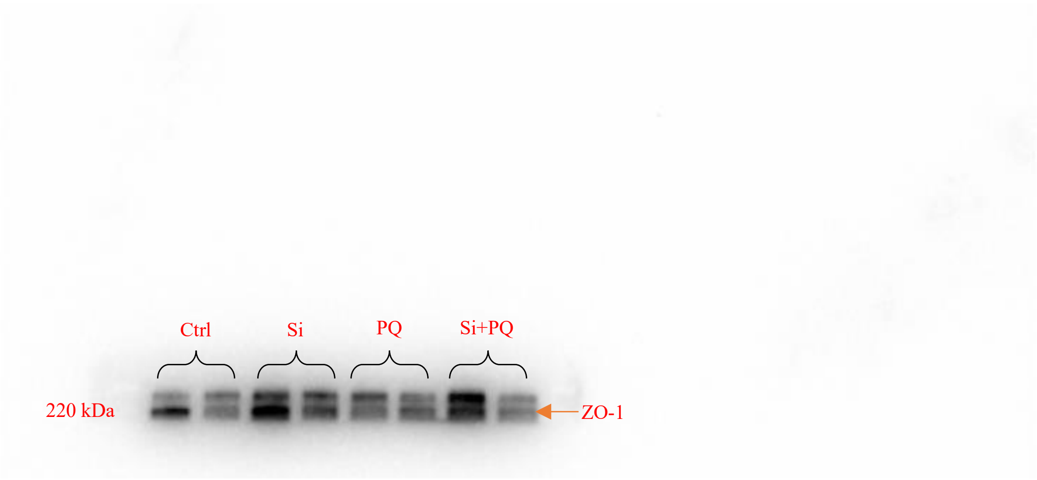

Fig. 3 Raw image of ZO-1 expression determined by Western blot (Repeat 3 and Repeat 4). Ctrl, basal diet group treated with saline; Si, silybin diet group treated with saline; PQ, basal diet group treated with paraquat; Si + PQ, silybin diet group treated with paraquat.

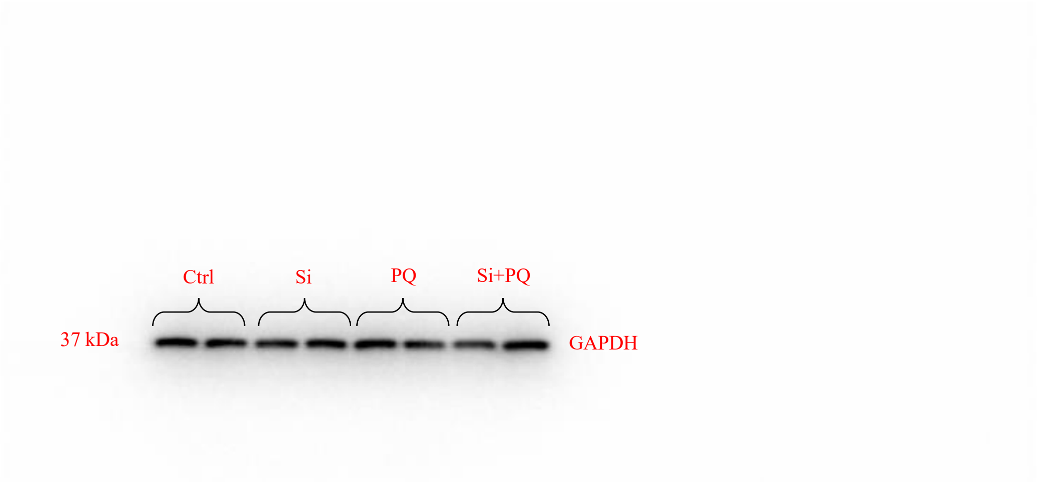

Fig. 4 Raw image of GAPDH expression determined by Western blot (Repeat 3 and Repeat 4). Ctrl, basal diet group treated with saline; Si, silybin diet group treated with saline; PQ, basal diet group treated with paraquat; Si + PQ, silybin diet group treated with paraquat.

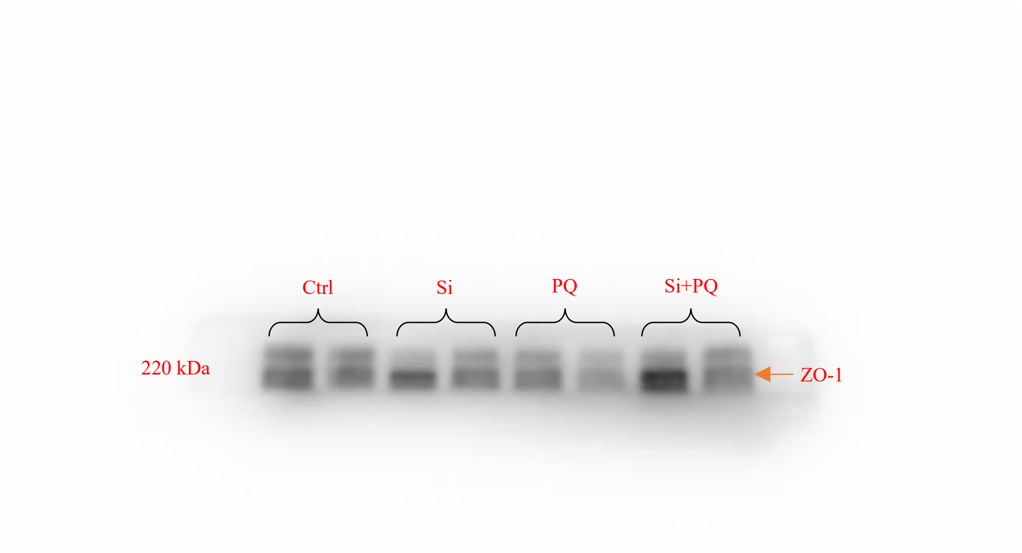

Fig. 5 Raw image of ZO-1 expression determined by Western blot (Repeat 5 and Repeat 6). Ctrl, basal diet group treated with saline; Si, silybin diet group treated with saline; PQ, basal diet group treated with paraquat; Si + PQ, silybin diet group treated with paraquat.

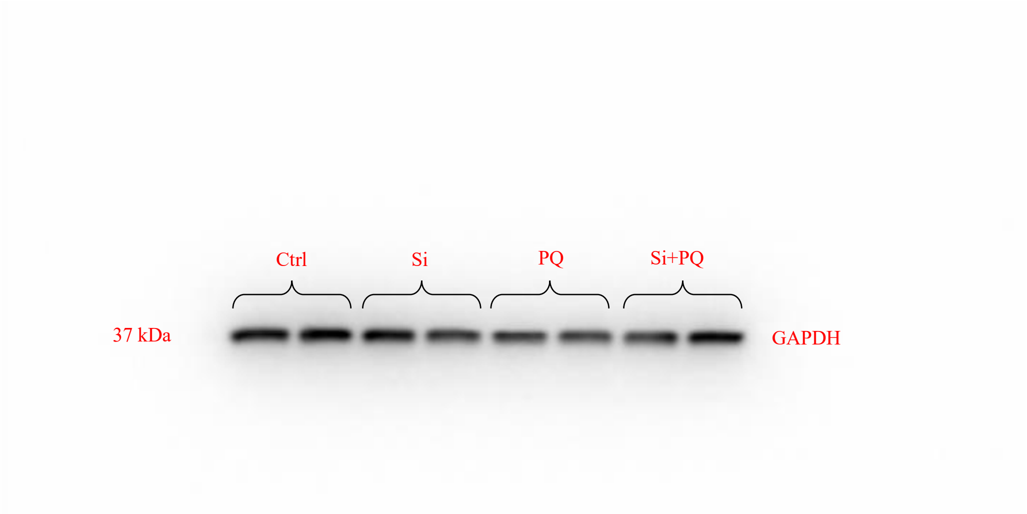

Fig. 6 Raw image of GAPDH expression determined by Western blot (Repeat 5 and Repeat 6). Ctrl, basal diet group treated with saline; Si, silybin diet group treated with saline; PQ, basal diet group treated with paraquat; Si + PQ, silybin diet group treated with paraquat.

For Occludin and Claudin 1:

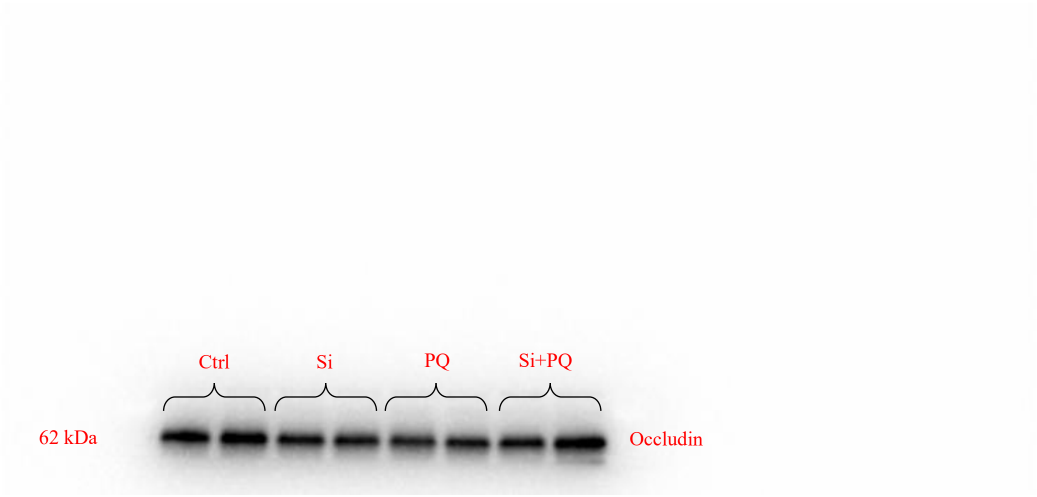

Fig. 1 Raw image of Occludin expression determined by Western blot (Repeat 1 and Repeat 2). Ctrl, basal diet group treated with saline; Si, silybin diet group treated with saline; PQ, basal diet group treated with paraquat; Si + PQ, silybin diet group treated with paraquat.

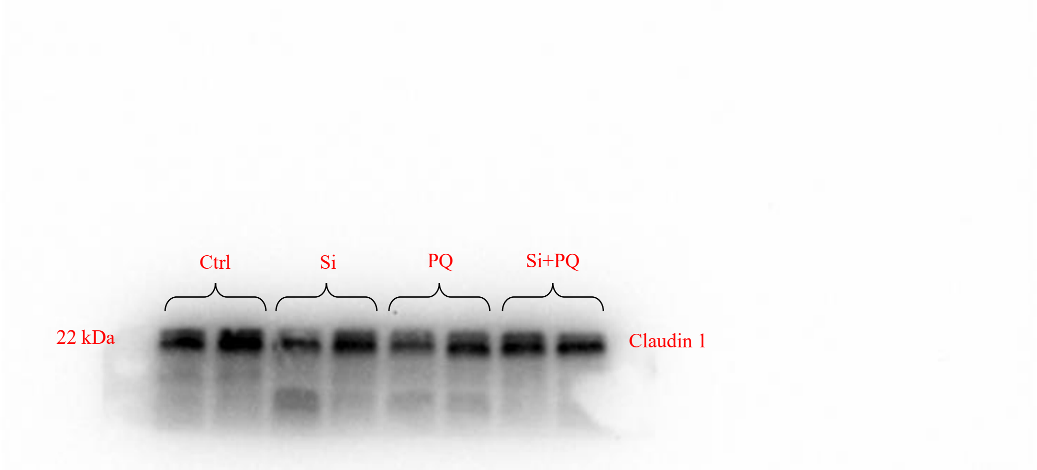

Fig. 2 Raw image of Claudin 1 expression determined by Western blot (Repeat 1 and Repeat 2). Ctrl, basal diet group treated with saline; Si, silybin diet group treated with saline; PQ, basal diet group treated with paraquat; Si + PQ, silybin diet group treated with paraquat.

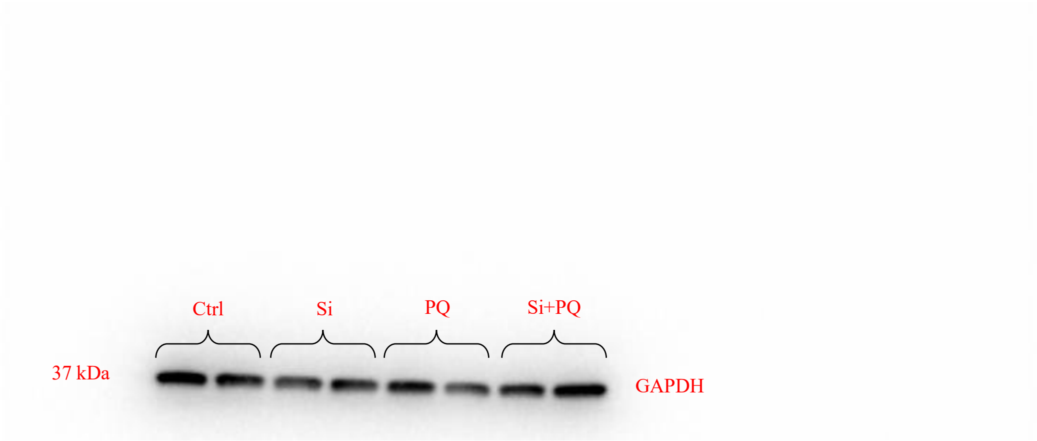

Fig. 3 Raw image of GAPDH expression determined by Western blot (Repeat 1 and Repeat 2). Ctrl, basal diet group treated with saline; Si, silybin diet group treated with saline; PQ, basal diet group treated with paraquat; Si + PQ, silybin diet group treated with paraquat.

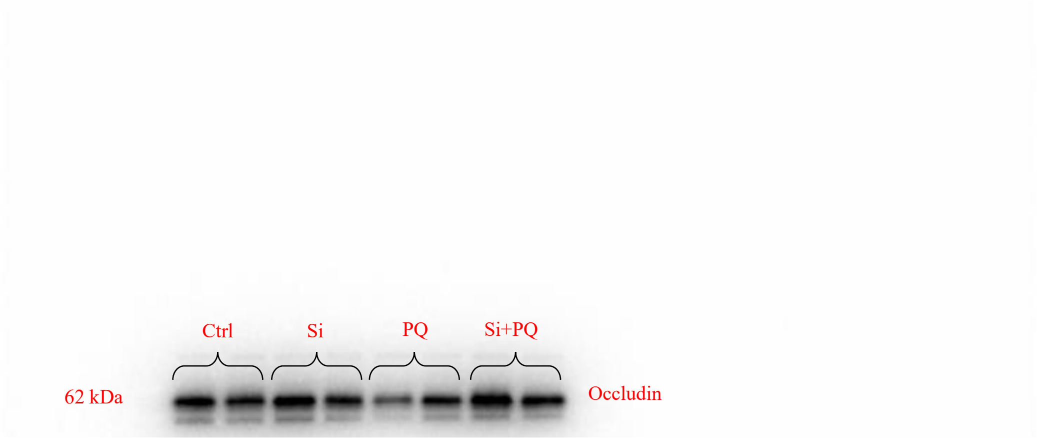

Fig. 4 Raw image of Occludin expression determined by Western blot (Repeat 3 and Repeat 4). Ctrl, basal diet group treated with saline; Si, silybin diet group treated with saline; PQ, basal diet group treated with paraquat; Si + PQ, silybin diet group treated with paraquat.

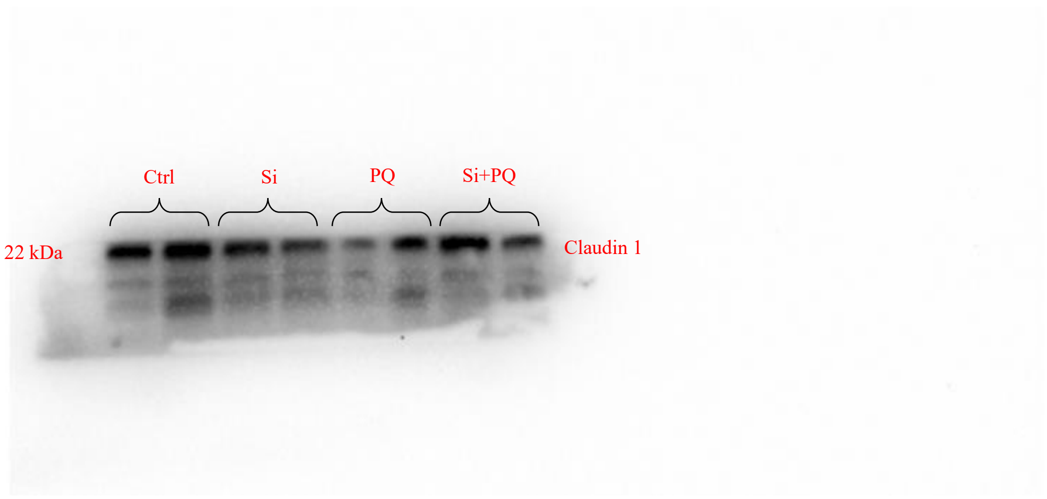

Fig. 5. Raw image of Claudin 1 expression determined by Western blot (Repeat 3 and Repeat 4). Ctrl, basal diet group treated with saline; Si, silybin diet group treated with saline; PQ, basal diet group treated with paraquat; Si + PQ, silybin diet group treated with paraquat.

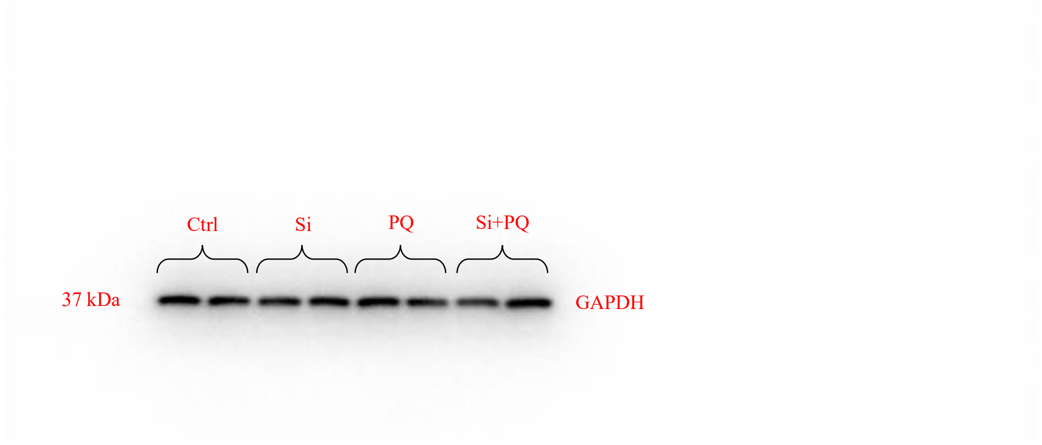

Fig. 6 Raw image of GAPDH expression determined by Western blot (Repeat 3 and Repeat 4). Ctrl, basal diet group treated with saline; Si, silybin diet group treated with saline; PQ, basal diet group treated with paraquat; Si + PQ, silybin diet group treated with paraquat.

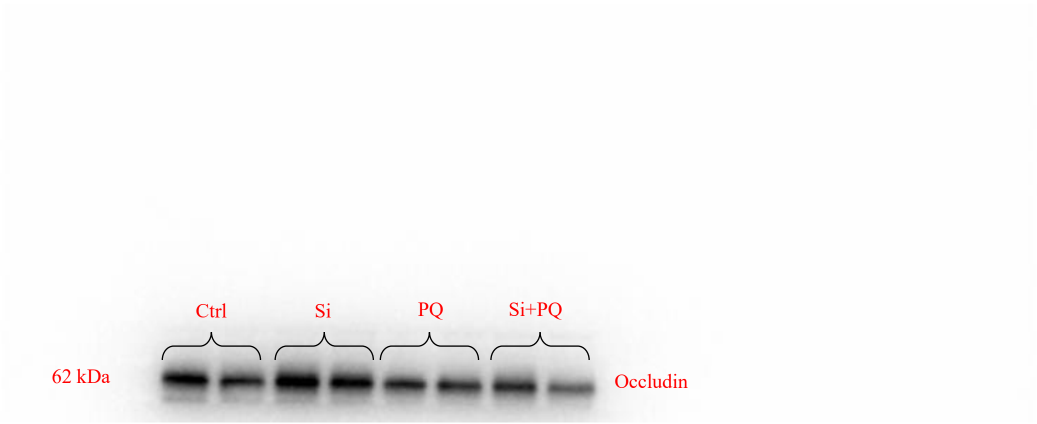

Fig. 7 Raw image of Occludin expression determined by Western blot (Repeat 5 and Repeat 6). Ctrl, basal diet group treated with saline; Si, silybin diet group treated with saline; PQ, basal diet group treated with paraquat; Si + PQ, silybin diet group treated with paraquat.

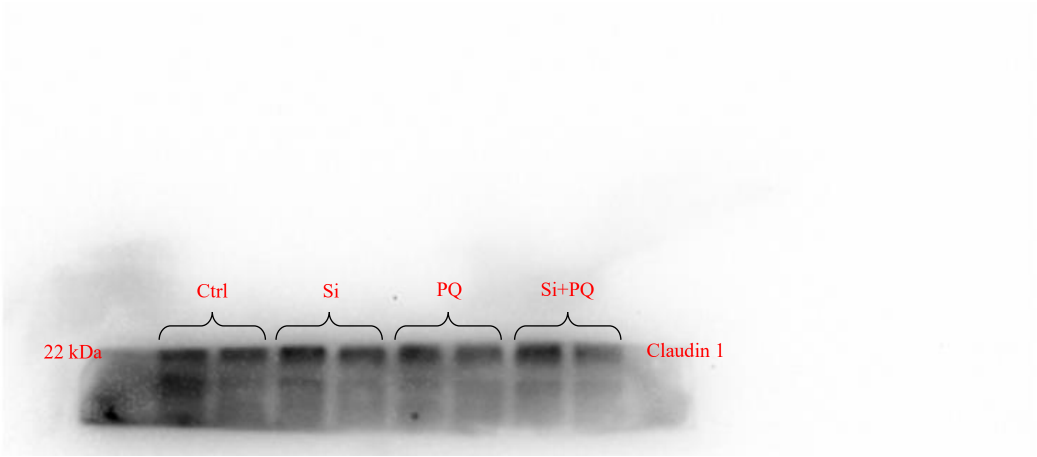

Fig. 8 Raw image of Claudin 1 expression determined by Western blot (Repeat 5 and Repeat 6). Ctrl, basal diet group treated with saline; Si, silybin diet group treated with saline; PQ, basal diet group treated with paraquat; Si + PQ, silybin diet group treated with paraquat.

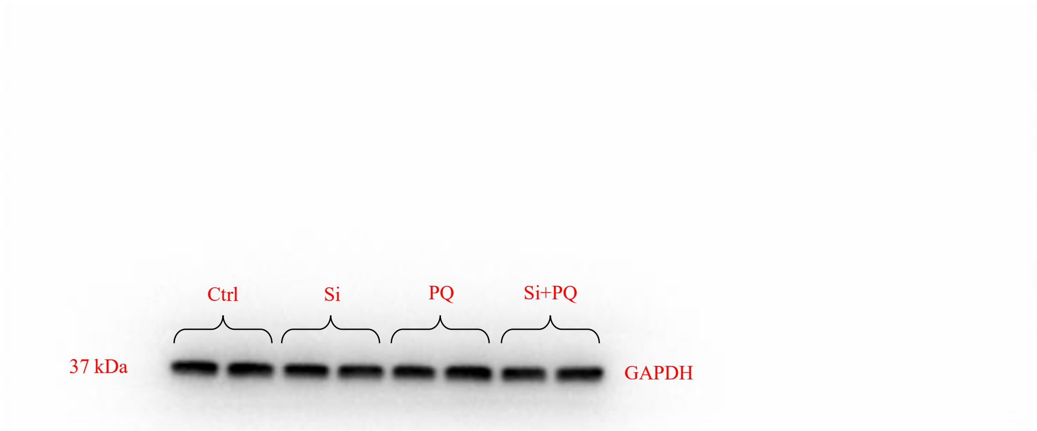

Fig. 9 Raw image of GAPDH expression determined by Western blot (Repeat 5 and Repeat 6). Ctrl, basal diet group treated with saline; Si, silybin diet group treated with saline; PQ, basal diet group treated with paraquat; Si + PQ, silybin diet group treated with paraquat.

For Cleaved caspase-3:

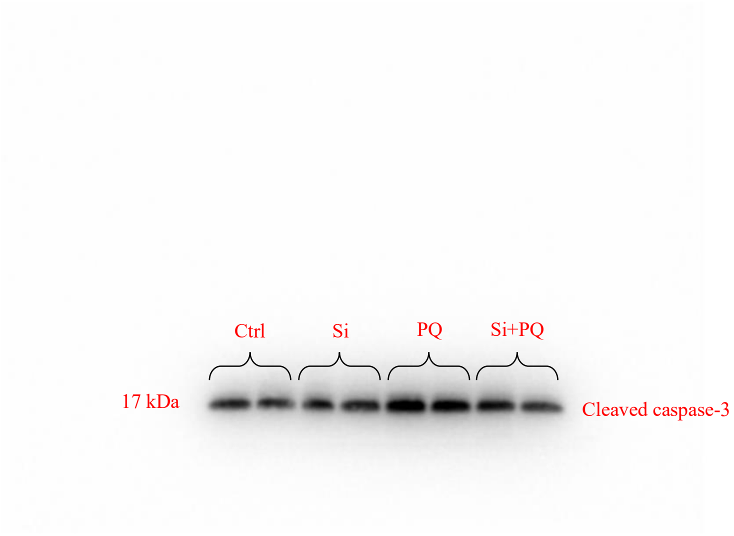

Fig. 1 Raw image of Cleaved caspase-3 expression determined by Western blot (Repeat 1 and Repeat 2). Ctrl, basal diet group treated with saline; Si, silybin diet group treated with saline; PQ, basal diet group treated with paraquat; Si + PQ, silybin diet group treated with paraquat.

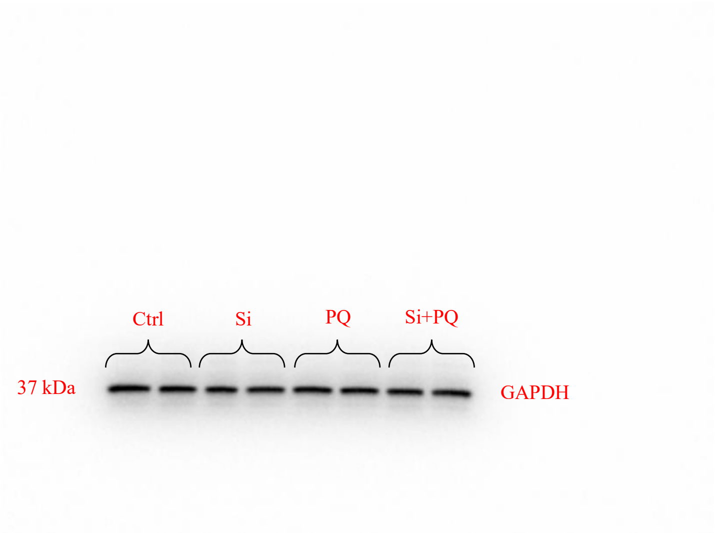

Fig. 2 Raw image of GAPDH expression determined by Western blot (Repeat 1 and Repeat 2). Ctrl, basal diet group treated with saline; Si, silybin diet group treated with saline; PQ, basal diet group treated with paraquat; Si + PQ, silybin diet group treated with paraquat.

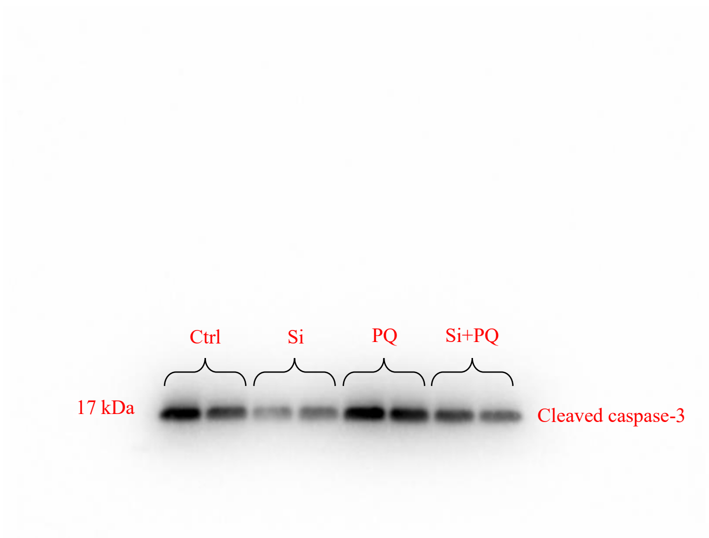

Fig. 3 Raw image of Cleaved caspase-3 expression determined by Western blot (Repeat 3 and Repeat 4). Ctrl, basal diet group treated with saline; Si, silybin diet group treated with saline; PQ, basal diet group treated with paraquat; Si + PQ, silybin diet group treated with paraquat.

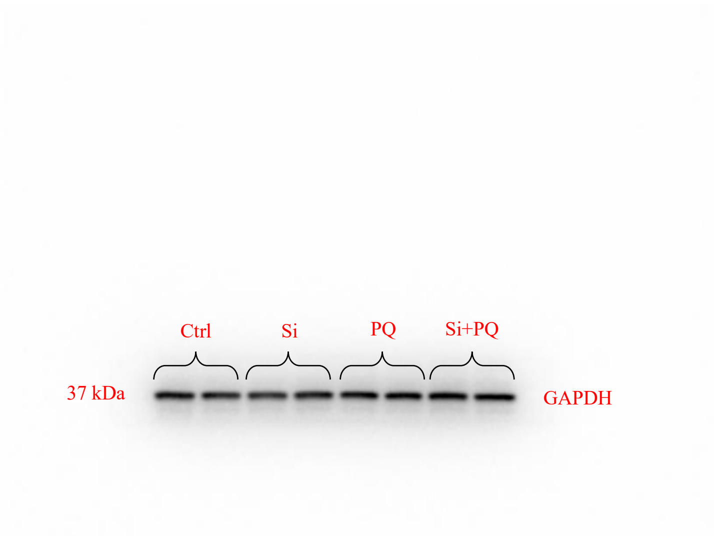

Fig. 4 Raw image of GAPDH expression determined by Western blot (Repeat 3 and Repeat 4). Ctrl, basal diet group treated with saline; Si, silybin diet group treated with saline; PQ, basal diet group treated with paraquat; Si + PQ, silybin diet group treated with paraquat.

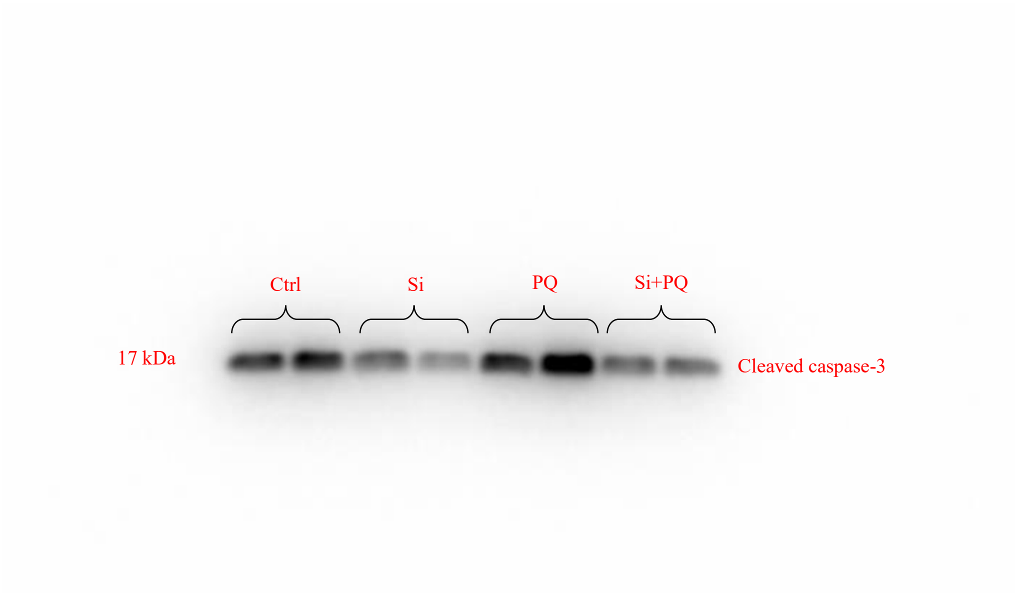

Fig. 5 Raw image of Cleaved caspase-3 expression determined by Western blot (Repeat 5 and Repeat 6). Ctrl, basal diet group treated with saline; Si, silybin diet group treated with saline; PQ, basal diet group treated with paraquat; Si + PQ, silybin diet group treated with paraquat.

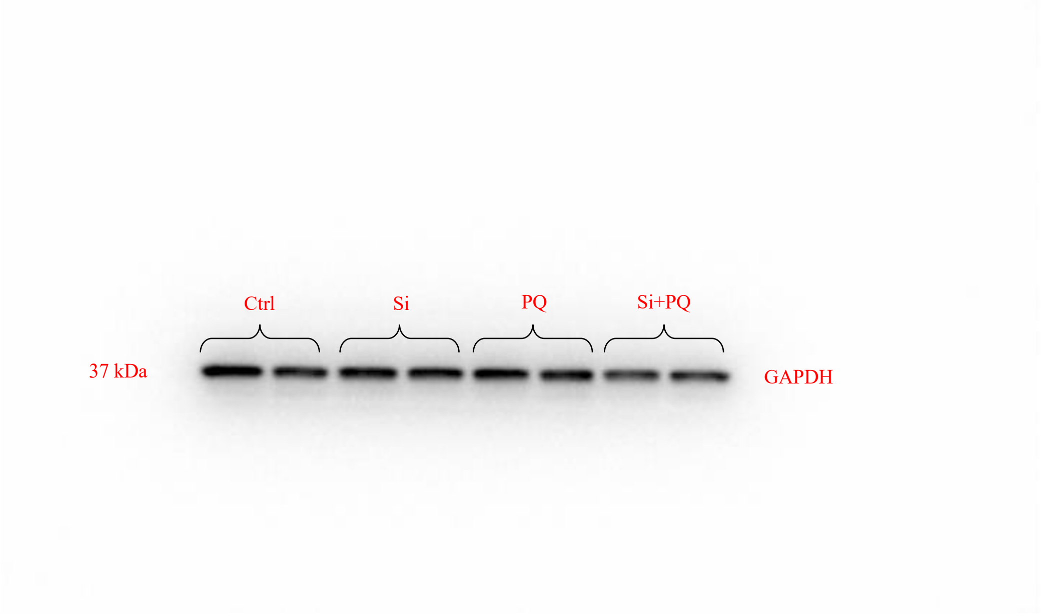

Fig. 6 Raw image of GAPDH expression determined by Western blot (Repeat 5 and Repeat 6). Ctrl, basal diet group treated with saline; Si, silybin diet group treated with

saline; PQ, basal diet group treated with paraquat; Si + PQ, silybin diet group treated with paraquat.

For Bcl-2 and Bax:

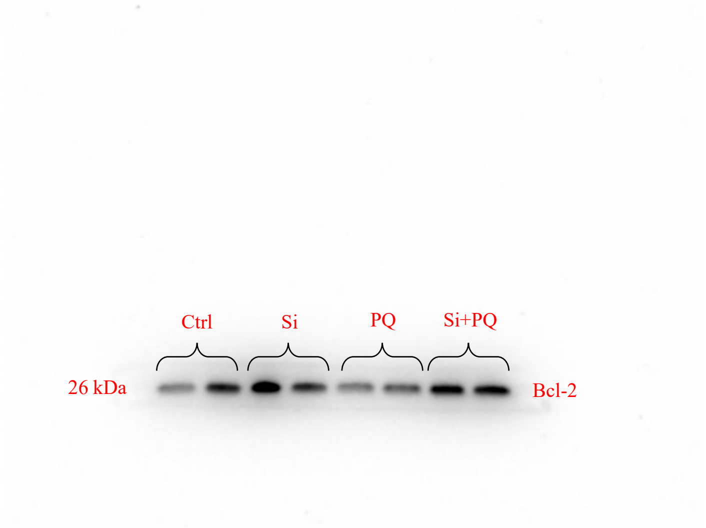

Fig. 1 Raw image of Bcl-2 expression determined by Western blot (Repeat 1 and Repeat 2). Ctrl, basal diet group treated with saline; Si, silybin diet group treated with saline; PQ, basal diet group treated with paraquat; Si + PQ, silybin diet group treated with paraquat.

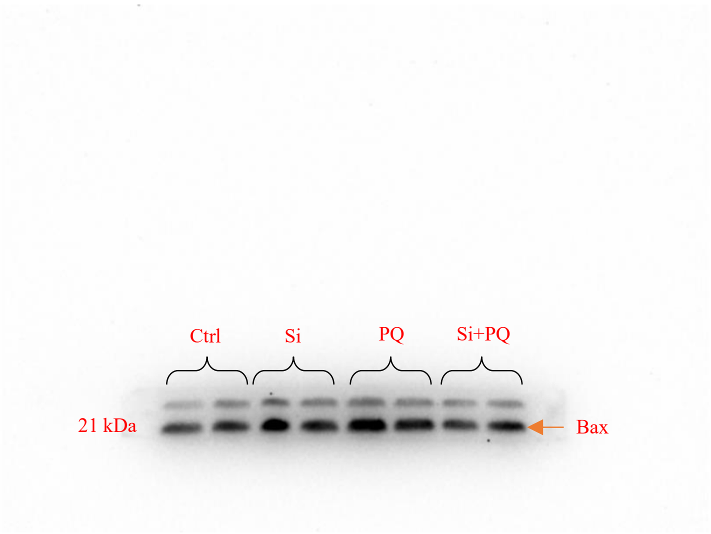

Fig. 2 Raw image of Bax expression determined by Western blot (Repeat 1 and Repeat 2). Ctrl, basal diet group treated with saline; Si, silybin diet group treated with saline; PQ, basal diet group treated with paraquat; Si + PQ, silybin diet group treated with paraquat.

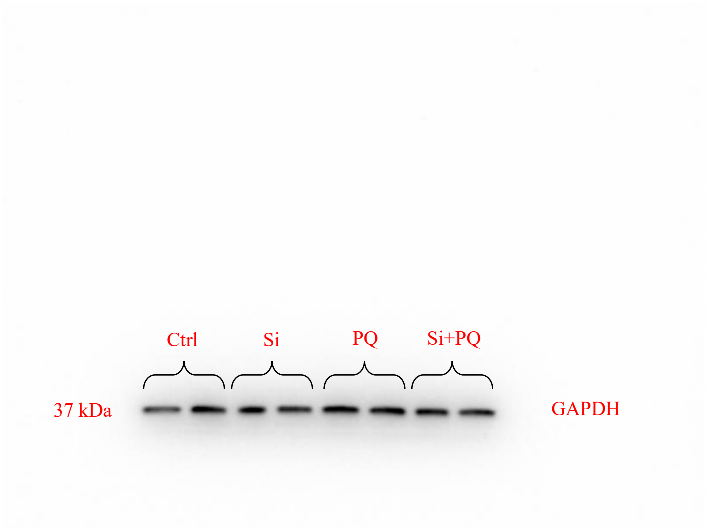

Fig. 3 Raw image of GAPDH expression determined by Western blot (Repeat 1 and Repeat 2). Ctrl, basal diet group treated with saline; Si, silybin diet group treated with saline; PQ, basal diet group treated with paraquat; Si + PQ, silybin diet group treated with paraquat.

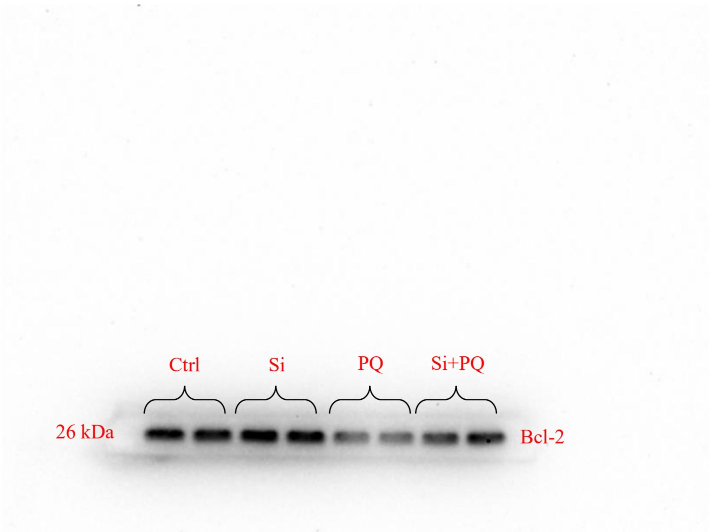

Fig. 4 Raw image of Bcl-2 expression determined by Western blot (Repeat 3 and Repeat 4). Ctrl, basal diet group treated with saline; Si, silybin diet group treated with saline; PQ, basal diet group treated with paraquat; Si + PQ, silybin diet group treated with paraquat.

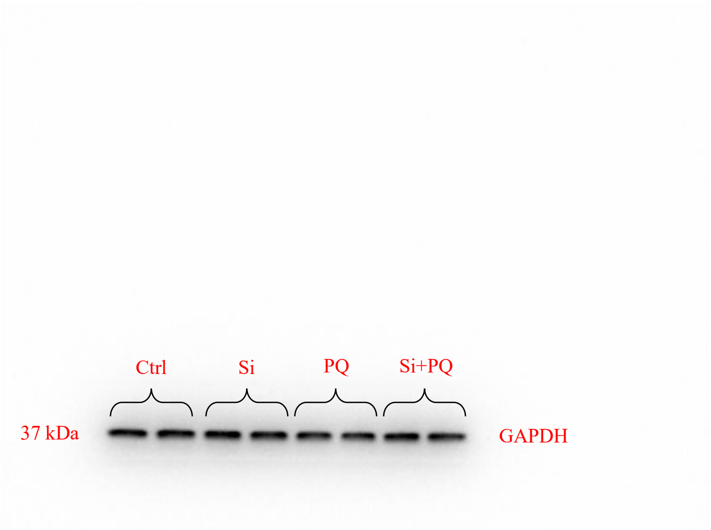

Fig. 5 Raw image of GAPDH expression determined by Western blot (Repeat 3 and Repeat 4). Ctrl, basal diet group treated with saline; Si, silybin diet group treated with saline; PQ, basal diet group treated with paraquat; Si + PQ, silybin diet group treated with paraquat.

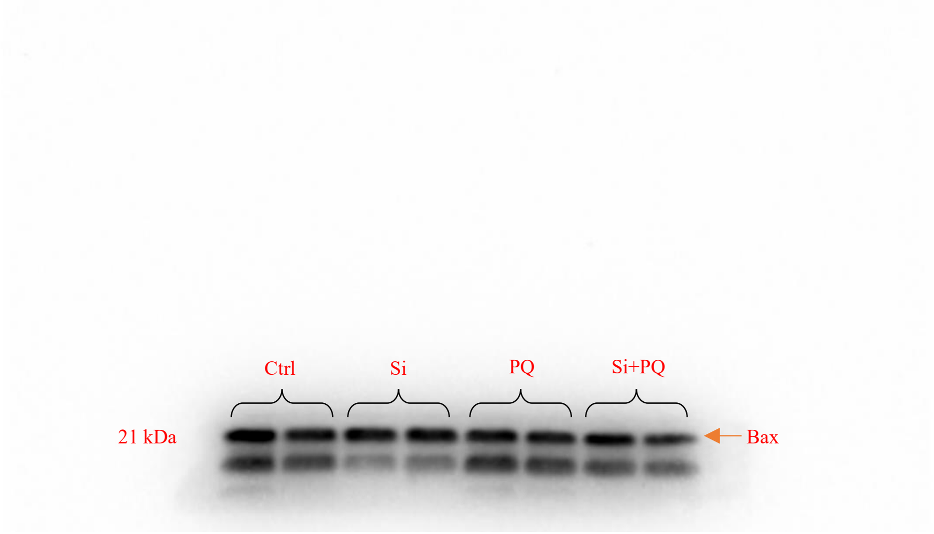

Fig. 6 Raw image of Bax expression determined by Western blot (Repeat 3 and Repeat 4). Ctrl, basal diet group treated with saline; Si, silybin diet group treated with saline; PQ, basal diet group treated with paraquat; Si + PQ, silybin diet group treated with paraquat.

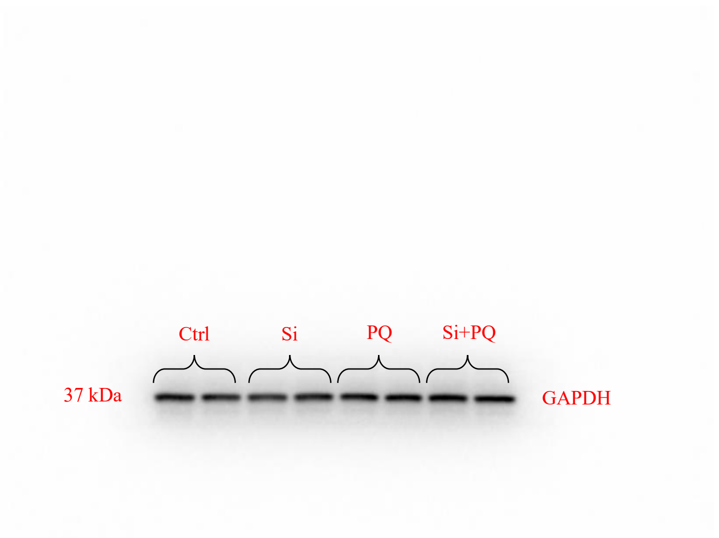

Fig. 7 Raw image of GAPDH expression determined by Western blot (Repeat 3 and Repeat 4). Ctrl, basal diet group treated with saline; Si, silybin diet group treated with saline; PQ, basal diet group treated with paraquat; Si + PQ, silybin diet group treated with paraquat.

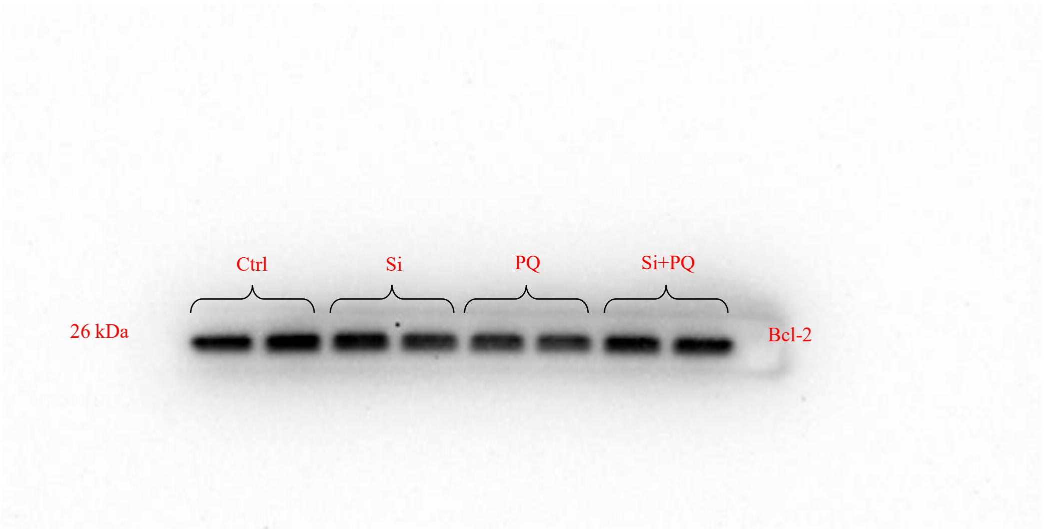

Fig. 8 Raw image of Bcl-2 expression determined by Western blot (Repeat 5 and Repeat 6). Ctrl, basal diet group treated with saline; Si, silybin diet group treated with saline; PQ, basal diet group treated with paraquat; Si + PQ, silybin diet group treated with paraquat.

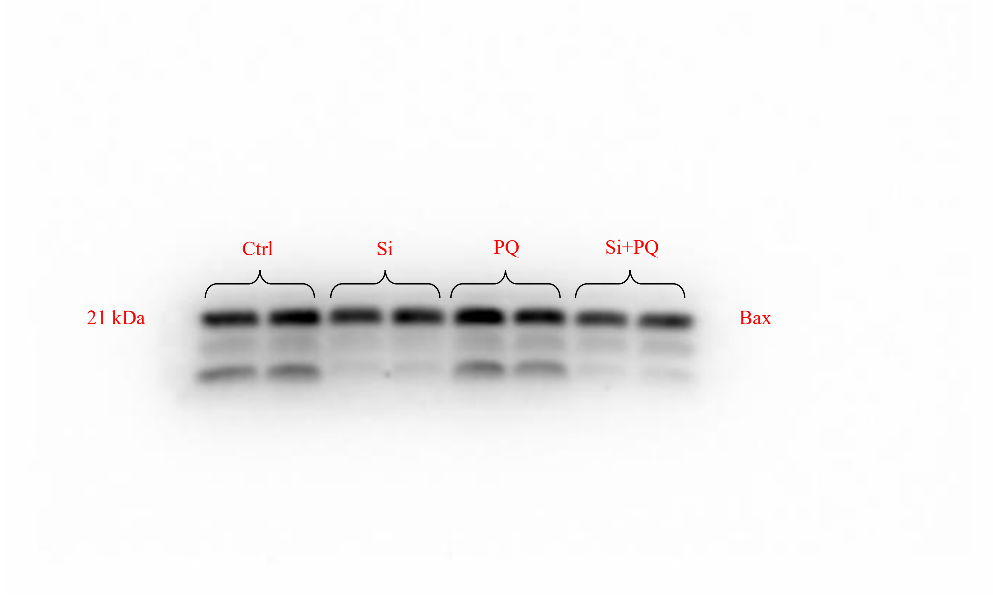

Fig. 9 Raw image of Bax expression determined by Western blot (Repeat 5 and Repeat 6). Ctrl, basal diet group treated with saline; Si, silybin diet group treated with saline; PQ, basal diet group treated with paraquat; Si + PQ, silybin diet group treated with paraquat.

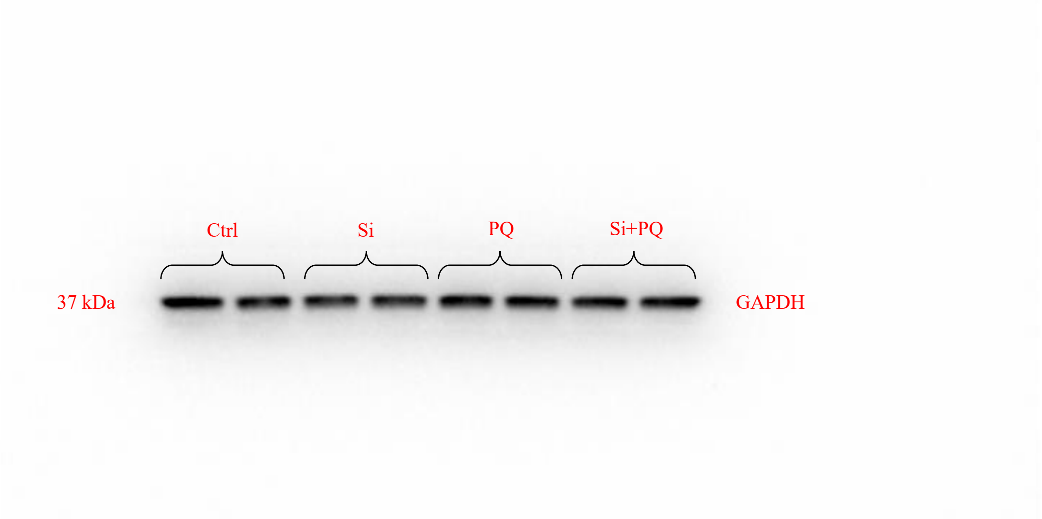

Fig. 10 Raw image of GAPDH expression determined by Western blot (Repeat 5 and Repeat 6). Ctrl, basal diet group treated with saline; Si, silybin diet group treated with saline; PQ, basal diet group treated with paraquat; Si + PQ, silybin diet group treated with paraquat.
